# Hydrological and environmental variables outperform spatial factors in structuring species, trait composition, and beta diversity of pelagic algae

**DOI:** 10.1002/ece3.3903

**Published:** 2018-02-14

**Authors:** Naicheng Wu, Yueming Qu, Björn Guse, Kristė Makarevičiūtė, Szewing To, Tenna Riis, Nicola Fohrer

**Affiliations:** ^1^ Department of Hydrology and Water Resources Management Institute for Natural Resource Conservation Kiel University Kiel Germany; ^2^ Aarhus Institute of Advanced Studies Aarhus University Aarhus C Denmark; ^3^ Department of Bioscience Aarhus University Aarhus C Denmark; ^4^ GFZ German Research Centre for Geosciences Section Hydrology Potsdam Germany; ^5^ Helmholtz Centre for Ocean Research Kiel (GEOMAR) Kiel Germany

**Keywords:** beta diversity, ecohydrological modeling, functional traits, lowland river, multiple stressors, pelagic algae, species composition

## Abstract

There has been increasing interest in algae‐based bioassessment, particularly, trait‐based approaches are increasingly suggested. However, the main drivers, especially the contribution of hydrological variables, of species composition, trait composition, and beta diversity of algae communities are less studied. To link species and trait composition to multiple factors (i.e., hydrological variables, local environmental variables, and spatial factors) that potentially control species occurrence/abundance and to determine their relative roles in shaping species composition, trait composition, and beta diversities of pelagic algae communities, samples were collected from a German lowland catchment, where a well‐proven ecohydrological modeling enabled to predict long‐term discharges at each sampling site. Both trait and species composition showed significant correlations with hydrological, environmental, and spatial variables, and variation partitioning revealed that the hydrological and local environmental variables outperformed spatial variables. A higher variation of trait composition (57.0%) than species composition (37.5%) could be explained by abiotic factors. Mantel tests showed that both species and trait‐based beta diversities were mostly related to hydrological and environmental heterogeneity with hydrological contributing more than environmental variables, while purely spatial impact was less important. Our findings revealed the relative importance of hydrological variables in shaping pelagic algae community and their spatial patterns of beta diversities, emphasizing the need to include hydrological variables in long‐term biomonitoring campaigns and biodiversity conservation or restoration. A key implication for biodiversity conservation was that maintaining the instream flow regime and keeping various habitats among rivers are of vital importance. However, further investigations at multispatial and temporal scales are greatly needed.

## 1 INTRODUCTION

Although rivers cover only 0.8% of the landmasses on the earth, they contain more than 6% of global species and are thus invaluable for biodiversity and ecosystem services (Altermatt, Seymour, & Martinez, 2013). They also act as conveyor belts of biodiversity information by dictating dispersal pathways (Deiner, Fronhofer, Mächler, Walser, & Altermatt, 2016), and thus, river ecosystems are a biodiversity hotspot. With arising from human‐mediated fast global change, water quality was degraded and the use of aquatic organisms in bioassessments became common in last decades. Studying the river organisms in relation to abiotic factors and identifying spatial patterns of biodiversity as well as their driving mechanisms have become a major trend of community ecology as basis for prioritizing global and regional conservation efforts (Myers, Mittermeier, Mittermeier, da Fonseca, & Kent, 2000; Wang, Pan, Soininen, Heino, & Shen, 2016). As the major primary producer, algae are increasingly being used as reliable environmental indicators in streams and rivers globally, especially in the context of recent international water framework directive policies such as EU Water Framework Directive (WFD; Hering et al., 2006; Lange, Townsend, & Matthaei, 2016; Wu et al., 2017) because they strongly respond to environmental changes (Larras et al., 2017; Stevenson, Pan, & van Dam, 2010; Wang, Li, et al., 2016).

The relationships between river algae and abiotic factors have been studied with a long history. Nevertheless, previous studies and biomonitoring campaigns focused mostly on local environmental variables such as nutrients (Kelly & Whitton, 1995; Lange, Liess, Piggott, Townsend, & Matthaei, 2011), pH, temperature (Çelekli, Öztürk, & Kapı, 2014; Wu, Schmalz, & Fohrer, 2011), and recently also spatial factors (Heino & MykrÄ, 2008; Rezende, Santos, Henke‐Oliveira, & Gonçalves, 2014; Tang, Niu, & Dudgeon, 2013; Tang, Wu, Li, Fu, & Cai, 2013; Wu, Cai, & Fohrer, 2014). By comparison, little attention has been paid to hydrological factors such as flow regime (Qu, Wu, Guse, & Fohrer, 2018), although many studies have shown that riverine algal communities are linked to flow velocity and discharge (Biggs, Smith, & Duncan, 1999; Jowett & Biggs, 1997; Munn, Frey, & Tesoriero, 2010; Riseng, Wiley, & Stevenson, 2004; Wu et al., 2010) and catchment wetness (Wu et al., 2016). Yet, a profound understanding on the interaction of hydrological variables and river organisms, specifically algae, is still missing.

In addition to species composition, ecologists have recently started investigating trait composition as it reflects the functional adaption of organisms to its environment (McGill, Enquist, Weiher, & Westoby, 2006; Soininen, Jamoneau, Rosebery, & Passy, 2016; Wang, Liu, Zhan, Yang, & Wu, 2017). Usually, traits are divided into two types: ecological traits (related to habitat preferences, such as pH, oxygen and temperature tolerance, and tolerance to organic pollution.) and biological traits (e.g., life history, physiological, behavioral, and morphological characteristics, such as reproductive strategies, motility, cell size, and life form). In comparison with traditional taxonomic indices, biological traits show greater consistency in their responses across temporal and spatial scales (Menezes, Baird, & Soares, 2010; Soininen et al., 2016) and furthermore give important insights into the mechanisms driving the community and ecosystem processes along the gradients of influential factors (Litchman & Klausmeier, 2008). Traits can furthermore serve to disentangle multiple interacting influential factors (Baattrup‐Pedersen, Göthe, Riis, & O'Hare, 2016; Lange, Townsend, & Matthaei, 2014). Trait‐based approaches have been used for different purposes in terrestrial plants (Grime, 1979; Tilman, 1980) and macroinvertebrate (Menezes et al., 2010), but only very recently been considered for freshwater algae (Lange et al., 2016; McGill et al., 2006; Tapolczai, Bouchez, Stenger‐Kovács, Padisák, & Rimet, 2016), particularly in phytoplankton studies (Colina, Calliari, Carballo, & Kruk, 2016; Padisák, Crossetti, & Naselli‐Flores, 2009; Reynolds, Huszar, Kruk, Naselli‐Flores, & Melo, 2002; Thomas, Kremer, & Litchman, 2016). Recent studies have shown the advantages of applying traits for biomonitoring of freshwater ecosystems and for biodiversity conservation (Di Battista, Fortuna, & Maturo, 2016; Lange et al., 2011; Litchman & Klausmeier, 2008; McGill et al., 2006; Menezes et al., 2010; Soininen et al., 2016). For instance, Soininen et al. (2016) concluded from a large‐scale study that trait distributions are driven primarily by the local environmental condition and less dependent on the spatial location, which makes them better suited for researches on global environmental change. However, the comparisons between species and trait composition in relation to abiotic factors at catchment scale, in particular multiple stressors, are still poorly documented.

Rivers are widely affected by a mixture of stressors caused by anthropogenic activities (Hering et al., 2015). Generally, they include flow regime alteration, diffuse, and point sources. For example, flow diversion due to dam construction can disrupt the river's natural connectivity and impede the cycling of organic matter, sediments, and nutrients from up‐ to downstream (Wu, Cai, & Fohrer, 2012). In addition, global land use and climate change pose additional stressors for rivers. The patterns of species composition in biological communities are governed by both local and spatial processes (Curry & Baird, 2015). Dispersal limitation creates spatial structure in assemblage composition because the probability of successful movement between locations is negatively related to the geographical distance between them. Spatial variables such as altitude or geographical location can play important and confounding roles determining the presence, absence, and abundance of the algal species and consequently influence the algae‐based bioassessment (Wu et al., 2014). One previous view of algae distributions was that they were ubiquitous and could disperse everywhere due to the immense population sizes, especially over a long time period (Fenchel & Finlay, 2004). If this theory was right, the similar algae species should be found at all places with similar environmental conditions, which was usually not the case leading to a large portion of variation explained by spatial factors (Smucker & Vis, 2011; Soininen, Paavola, & Muotka, 2004). Thus, it is difficult to determine whether an absent species is due to the unallowable environmental conditions or it has not dispersed to that location. Studying spatial geographical influences on algal composition is therefore a fundamental step in describing ecological patterns, making biomonitoring more robust, which is essential for sustainable management (Smucker & Vis, 2011). Nevertheless, the current biomonitoring using algae often focuses on the local environmental conditions with seldom regarding processes operating at larger spatial scales.

**Figure 1:**
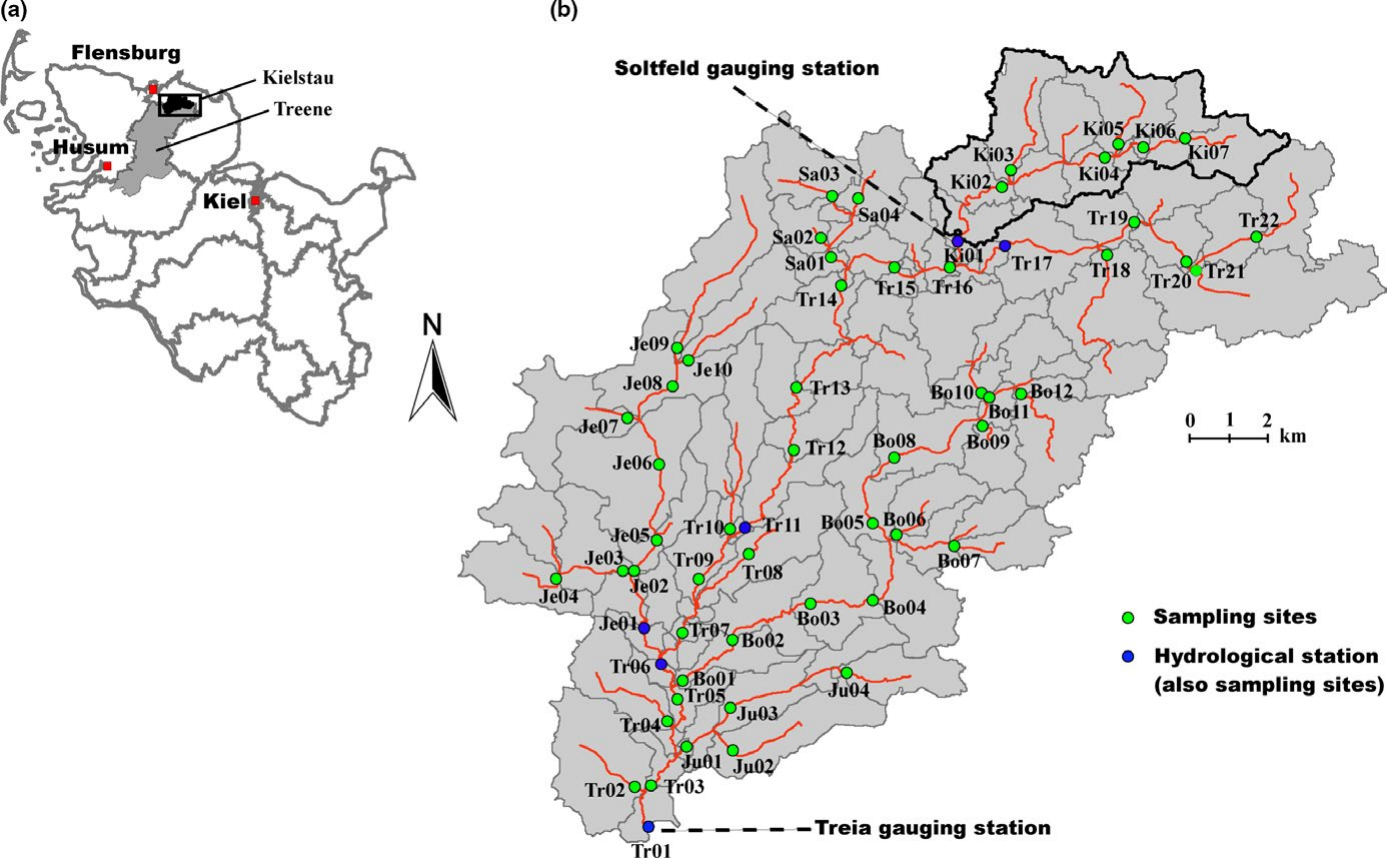
The location of six hydrological stations and sampling sites of the Treene catchment (b) in Schleswig‐Holstein state (a) of Germany. Subbasins of Treene as derived by the ecohydrological model SWAT (Soil and Water Assessment Tool) are shown too

The purpose of this study was to assess the influence of different factors (e.g., hydrological variables, local environmental variables, and spatial factors) on shaping species composition, trait composition, and beta diversities of riverine pelagic algae communities in a German lowland catchment (Figure 1). We had two main questions for this research: (i) How much do hydrological variables contribute to variations of species and trait compositions compare to local environmental and spatial variables? (ii) What are the major drivers of the species, trait composition, and beta diversities of pelagic algae communities? The hypotheses were that (i) hydrological, environmental, and spatial variables interacted to determine species composition, trait composition, and beta diversity of pelagic algae, (ii) hydrological variables would be a key driver of species composition, trait composition, and beta diversity, (iii) trait distributions are less dependent on historic (i.e., spatial) variables than species composition.

## 2 MATERIALS AND METHODS

### 2.1 Description of the study area

The Treene catchment with a basin area of 481 km^2^ is located in northern Germany as a part of a lowland area (Figure 1). Sandy, loamy, and peat soils are characteristic for this area. Land use is dominated by agriculture and pasture. Around 50% of the area is covered by arable land and ~30% by winter pasture (Guse, Pfannerstill, & Fohrer, 2015). The major tributaries are Bondenau (Bo), Kielstau (Ki), Bollingstedter Au (Bo), Jerrisbek (Je), Juebek (Ju), and Sankermarker See (Sa). It is in a temperate climate zone, influenced by marine climate, with mild temperature and high precipitation in winter, and the maximum elevation gradient is 76 m. There are several lakes (top three from size: Sueden See: 0.64 km^2^, Sankermarker See: 0.56 km^2^, and Winderatter See: 0.24 km^2^) in the catchment, mainly located in the upstream areas of the river. As a nested subcatchment of the Treene, the Kielstau catchment (50 km^2^; Figure 1) has been appointed to an UNESCO Ecohydrological Demonstration Site in the year 2010 (Fohrer & Schmalz, 2012). The Soltfeld gauging station (at the outlet of the Kielstau catchment) and Treia gauging station (at the outlet of Treene catchment) are a part of the official gauging network of the Federal State Schleswig‐Holstein (Figure 1b). In addition, four more spatially distributed hydrological stations with continuous daily discharge time series were used for this study as shown in Figure 1b. The Treene catchment was selected because of the reliability of the well‐proven hydrological SWAT model (Guse, Kail, et al., 2015; Guse, Pfannerstill, et al., 2015), which enables to simulate long‐term discharges at different sampling sites. These conditions, which are rare in previous studies, are ideal to apply and test our hypotheses.

### 2.2 Sampling methods and primary procedures

Field surveys were carried out in the mainstream and its tributaries in December 2014. We visited 59 sampling sites and abbreviated the sites according to each subbasin they were located in. Pelagic algae were collected using plankton net with a mesh size of 20 μm. A known volume of water (10–30 L, depending on site) was filtered and fixed immediately by neutral Lugol's solution. Algae samples were concentrated to 25 ml for further processing after natural sedimentation in the laboratory.

Simultaneously, at each sampling point, water temperature (WT), pH, electric conductivity (EC), and dissolved oxygen (DO) of the surface water were measured in situ using Portable Meter (WTM Multi 340i and WTW Cond 330i, Germany). Besides, river width, depth, and velocity were surveyed at the sampling points (velocity—using FlowSens Single Axis Electromagnetic Flow Meter, Hydrometrie, Germany).

Concurrently, water samples were taken in two precleaned plastic bottles (500 ml each) for water chemistry measurement in the laboratory. In the laboratory, water samples were partially filtrated through GF/F glass microfiber filter (Whatmann 1825‐047) for measurements of phosphate‐phosphorus (PO_4_‐P), ammonium‐nitrogen (NH_4_‐N), nitrate‐nitrogen (NO_3_‐N), nitrite‐nitrogen (NO_2_‐N), chloride (Cl^−^), and sulfate (${\text{SO}}_{4}^{2-})$ according to the standard methods DEV (Deutsche Einheitsverfahren zur Wasser‐, Abwasser‐ und Schlammuntersuchung). The concentrations of total phosphorus (TP) were measured with unfiltrated water samples. PO_4_‐P and TP were measured using the ammonium molybdate spectrophotometric method (at 880 nm; DIN 1189). We used Nessler's reagent colorimetric method (DIN 38 406‐E5‐1) to measure NH_4_‐N concentrations at 690 nm. NO_3_‐N, NO_2_‐N, Cl^−^, and (${\text{SO}}_{4}^{2-})$ were measured by an ion chromatography method (DIN 38 405‐D19). Dissolved inorganic nitrogen (DIN) was defined as the sum of NO_2_‐N, NO_3_‐N, and NH_4_‐N. Total suspended solids (TSS) were measured according to standard operating procedure for total suspended solid analysis (US Environmental Protection Agency, 1997). Inorganic carbon (IC), dissolved total carbon (DTC), and dissolved organic carbon (DOC) were measured with a DIMA‐TOC‐100 total organic carbon analyzer, according to infrared spectroscopy method (Dimatec Analysentechnik GmbH, Germany).

### 2.3 Algae preparation and identification

For the soft algae (nondiatom) identification, algae were counted with optical microscope (Nikon Eclipse E200‐LED, Germany) at ×400 magnifications in a Fuchs–Rosenthal chamber. The counting unit was individual (unicell), and at least, 300 units were counted for each sample. Taxonomic identification of species was carried out according to Hu and Wei (2006), Burchardt (2014).

To identify diatoms, permanent diatom slides were prepared after oxidizing the organic material by hydrogen peroxide method (30% H_2_O_2_ solution) and mounted on slides using Naphrax (Northern Biological supplies Ltd., UK, R1 = 1.74). A minimum of 300 valves was counted for each sample using a Zeiss Axioskop microscope at 1,000× under oil immersion. Diatoms were identified to the lowest taxonomic level possible (mainly species level) according to following key books (Bey & Ector, 2013; Lange‐Bertalot, 2000a, 2000b, 2005, 2007; Round, Crawford, & Mann, 1990; Simonsen, 1987), Hofmann (Hofmann, Werum, & Lange‐Bertalot, 2011), and Bak (Bąk et al., 2012). Algae densities were expressed as cells/L.

### 2.4 Biotic datasets

We used both traditional taxonomic composition and a functional perspective based on species traits composition.

1. Species composition (Sp): inclusion of all observed 327 algal species with their relative abundances.
2. Trait composition (Tr): We assigned 327 algal species to different functional traits: cell sizes (pico, nano, micro, meso, macro, and large), guilds (low profile, high profile, motile, and planktonic guild), life form (colonial, filamentous, flagellate, and unicellular), ecomorphology (combination between cell sizes and guilds + life form), nitrogen fixation species, reproductive strategies (fission and fragmentation), and spore formation (no spore, akinetes, oospores, and zygospores) (Appendix S1). Traits with medians of 0 were eliminated because they would prevent the further statistical analyses, and thus, 44 traits were retained for final Tr dataset.
3. Beta diversities (ß): To calculate the pairwise dissimilarities, we used the Bray–Curtis similarity index on Sp and Tr separately (i.e., Spß_BRAY_ and Trß_BRAY_), as this index takes into account differences in abundances and emphasizes dominant species/trait (Magurran, 2004). Similarly, we also employed Jaccard similarity index on Sp and Tr, respectively (i.e., Spß_JACC_ and Trß_JACC_).

### 2.5 Abiotic datasets

Three abiotic datasets were formed.

1. Hydrological variables (Hv): Except for in situ measured width, water depth, and flow velocity at the sampling point, long‐term flow discharges (2010–2016) of each sampling site were simulated by the ecohydrological SWAT model (Soil and Water Assessment Tool; Arnold, Srinivasan, Muttiah, & Williams, 1998). The SWAT model is a semidistributed model which provides daily outputs of a large set of hydrological variables for each subbasin. In this case study, the Treene catchment was subdivided into 108 subbasins (Figure 1), which also covered the tributaries of the Treene (Guse, Reusser, & Fohrer, 2014). Thus, the spatially distributed model results consider the spatial heterogeneity in the catchment. Three input maps were implemented in the SWAT model setup: a digital elevation model, a land use map, and a soil map (Guse, Pfannerstill, et al., 2015). To obtain reliable spatially distributed model results, a multisite calibration approach was selected and six hydrological stations were included in the calibration procedure with the aim to obtain good model results for all stations (Guse, Pfannerstill, et al., 2015). We used a calibration period from 2001 to 2005 and validated the model from 2006 to 2016 for discharge. As the measurements at one hydrological station ended in 2014, only five stations were used in the model validation. To assess the model performance, three typical performance measures namely Nash–Sutcliffe efficiency (NSE), percent bias (PBIAS), and the root mean square error deviation (RSR) were used. The modeled discharge shows a good matching with the measured data, in particular under consideration of the multisite approach with joint model performance estimation for six hydrological stations (for details see Guse, Kail, et al. 2015; Guse, Pfannerstill, et al., 2015). Based on the well‐performing model, daily model results for the investigation period of this study were provided for all subbasins with sampling points. In order to obtain reliable results, always the next subbasin outlet was used for each sampling point under consideration of the river network. Then, we calculated the different hydrological indices according to Olden and Poff (Olden & Poff, 2003), which mainly included magnitude of flow events, frequency of flow events, rate of change in flow events, and *in situ* measurement (details see Appendix S2). Finally, 11 hydrological variables were selected after excluding the ones with significant multicollinearity (Table 1, Appendices S2 and S3).
  **Table 1:** Summary of hydrological (Hv), environmental (Ev), and spatial (Sv) variables with their codes and descriptions in this study

Variables					
Code	Unit	Description	Mean	Min	Max
Hv		Hydrological variables			
Hv01	m^3^/s	Discharge at the sample day	2.27	0.01	18.30
Hv12	–	Skewness of 3 days' ahead discharge (including the sampling day)	0.32	−1.73	1.73
Hv13	–	Skewness of 3 days' ahead discharge (excluding the sampling day)	0.90	−1.69	1.73
Hv20	–	Skewness of 7 days' ahead discharge (including the sampling day)	0.95	−0.22	2.44
Hv21	–	Skewness of 7 days' ahead discharge (excluding the sampling day)	0.84	−1.58	2.64
Hv36	–	Skewness of 30 days' ahead discharge (including the sampling day)	1.14	−0.31	3.28
Hv40	D	Low flood pulse count in the past 14 days	4.71	0.00	14.00
Hv45	D	High flood pulse count in the past 30 days	4.31	0.00	12.00
Hv54	–	Rate of change (i.e., slope) in the last 3 days before the sampling day	0.18	−0.01	1.50
Hv55	–	Rate of change (i.e., slope) in the last 7 days before the sampling day	−0.04	−0.31	0.09
VELO	m/s	Flow velocity at the sampling point	0.98	0.00	10.24
Ev		Environmental variables			
WT	°C	Water temperature	5.69	0.20	8.40
PH	–	pH	7.49	6.74	9.73
DO	mg/L	Dissolved oxygen	9.49	4.61	12.30
TP	mg/L	Total phosphorus	0.22	0.06	0.63
PO_4_	mg/L	Orthophosphate‐phosphorus (PO_4_‐P)	0.08	0.01	0.34
NH_4_	mg/L	Ammonium‐nitrogen (NH_4_‐N)	0.31	0.03	1.43
NO3	mg/L	Nitrate‐nitrogen (NO_3_‐N)	3.55	1.03	8.43
NO_2_	mg/L	Nitrite‐nitrogen (NO_2_‐N)	0.02	0.00	0.05
CL	mg/L	Chloride (Cl^−^)	24.92	14.20	41.70
SO_4_	mg/L	Sulfate ((${\text{SO}}_{4}^{2-})$ )	31.82	12.90	73.10
TSP	mg/L	Total suspended particulates	12.08	2.60	46.28
DTC	mg/L	Dissolved total carbon	41.59	25.60	70.40
DOC	mg/L	Dissolved organic carbon	10.45	−0.15	29.50
AGRL	%	Agricultural Land‐Generic (%)	51.83	15.04	79.65
FRSD	%	Deciduous forest (%)	2.23	0.01	9.89
FRSE	%	Evergreen forest (%)	1.02	0.02	9.02
FRST	%	Forests mixed (%)	2.46	0.00	13.47
FR	%	Forest in total (%)	5.71	0.86	15.13
RNGE	%	Rangeland (%)	0.70	0.00	4.33
UIDU	%	Industrial (%)	4.20	2.98	8.41
URLD	–	Residential‐Low Density	0.43	0.00	3.98
UR	–	Residential in total	5.65	1.75	12.26
WATR	%	Water (%)	1.71	0.62	5.42
WETL	%	Wetlands (%)	1.01	0.00	7.19
WPAS	%	Winter pasture (%)	29.18	7.22	70.97
Sv		Spatial variables			
X	N	Latitude	54.64	54.51	54.74
Y	E	Longitude	9.43	9.27	9.67
PCNM1	–	Principal coordinates of neighborhood matrix1	0.00	−0.14	0.23
PCNM3	–	Principal coordinates of neighborhood matrix3	0.00	−0.29	0.24
PCNM6	–	Principal coordinates of neighborhood matrix6	0.00	−0.34	0.26
PCNM7	–	Principal coordinates of neighborhood matrix7	0.00	−0.31	0.28
PCNM10	–	Principal coordinates of neighborhood matrix10	0.00	−0.33	0.26
PCNM11	–	Principal coordinates of neighborhood matrix11	0.00	−0.24	0.55

Variables indicating significant multicollinearity (with variance inflation factor >10 and Spearman correlation coefficient ≥0.75) are excluded. For spatial variables, only the variables after forward selection are shown here (see also Table 2).
2. Environmental variables (Ev): Ev includes in situ and laboratory‐measured physicochemical variables (see above). Furthermore, land use data were obtained from Schleswig‐Holstein State Bureau of Surveying and Geo‐information (LVERMGEO‐SH, 2012). Land use analysis was performed via GIS processing. Watershed area upstream from each sampling site was determined, and land use within this area was considered as the land use affecting the sampling site. A total of 25 variables were retained after excluding the ones with significant multicollinearity (Table 1, Appendix S4).
3. Spatial variables (Sv): Except for the coordinates (X: latitude, Y: longitude), Moran's eigenvector maps were used to generate spatial variables representing geographical positions and dispersal across the rivers. This method is a powerful approach able to detect spatial structures of varying scale in response to data and more flexible than other eigenvector‐based approaches for irregular sampling design (Tang, Niu, et al., 2013; Tang, Wu, et al., 2013), as the case in our study. In brief, this method proceeds as follows: (i) a geographical distance matrix as Euclidean distance between each pair of sampling sites was calculated using the *earth.dist* function in the package *fossil* in R (version 3.3.2). (ii) Principal coordinates of neighborhood matrix (PCNM) analysis based on the geographical distance was used to compute spatial variables (i.e., historic factors) representing geographical positions through the *pcnm* function in R package *vegan* (version 2.4‐2). The generated eigenvectors were considered as spatial variables (i.e., PCNMs), which could reflect unmeasured broadscale variation in the modern environment or historic factors, for example, natural dispersal‐generated patterns demonstrating internal local‐scale dispersal dynamics or regional‐scale migration history (Svenning, Baktoft, & Balslev, 2009). PCNMs with large eigenvalues and small code represent broadscale spatial pattern, while the smaller eigenvalues with large code represent fine‐scale patterns. PCNMs are commonly used to describe species dispersal processes (Curry & Baird, 2015). Usually, only PCNMs with positive eigenvalues are retained as spatial explanatory variables (Tang, Niu, et al., 2013; Tang, Wu, et al., 2013). Among the 58 PCNMs generated, eigenvalues of PCNM components 1–37 were positive, and thus, 39 variables (including X, Y) were used in the following analyses (Table 1, Appendix S5).

### 2.6 Data analysis

All analyses were performed with the R software (version 3.3.2, R Development Core Team 2017).

To explore the potential impacts of hydrological variables on trait and species compositions (*question i*), the following preliminary data analyses were conducted. Firstly, trait and species composition with relative abundance (0–100%) were Hellinger‐transformed (using function *decosdtand* in R package *vegan*), respectively, in order to reduce the weight of abundant species/trait while preserves Euclidean distances between samples in the multidimensional space. Secondly, the variables in abiotic datasets (Hv, Ev, and Sv) with significant multicollinearity (with variance inflation factor >10 and Spearman's rank correlation coefficient |*r*| ≥ .75) were excluded (details see also above). A preliminary detrended correspondence analysis (DCA, using function *decorana* in R package *vegan*) on the Hellinger‐transformed trait and species data produced a longest gradient length of 2.03 and 4.82 along the first axis, suggesting that redundancy analysis (RDA) and canonical correspondence analysis (CCA) were appropriate for Tr and Sp, respectively (Lepš & Šmilauer, 2003). We performed RDA using the *rda* function and CCA using *cca* function and tested the significance using the *anova* function. Only if it was significant, a forward selection could be proceeded to get a parsimonious model with two stopping criteria: significance level and the adjusted coefficient of determination (Adj *R* ^2^) of the global model (Blanchet, Legendre, & Borcard, 2008). Forward selection was performed by the *forward.sel* function in R package *packfor*. The selected variables were then used as explanatory variables for the following variation partitioning analysis using *varpart* function R package *vegan* (version 2.4‐2).

Next, we ran Mantel tests in order to examine the changes in trait and species composition along hydrological, environmental, and spatial gradients (*question ii*). The Mantel test has been utilized as a distance‐based approach to study community beta diversities in relation to distance matrices (Teittinen, Kallajoki, Meier, Stigzelius, & Soininen, 2016; Wang et al., 2012). The significance of this distance–decay relationship, which measures how dissimilarity decays with increasing distance between pairwise sites, was determined using Mantel test with 9,999 permutations. In brief, the Mantel statistic *r* (range −1 to 1) is a correlation between two dissimilarities or distance matrices. We first constructed dissimilarity matrices for biotic data (i.e., beta diversities, Spß_BRAY_, Trß_BRAY_, Spß_JACC_, and Trß_JACC_, for details see above) and Euclidean distances separately for the hydrological, environmental, and spatial variables (i.e., Hvdis, Evdis, and Svdis). In addition to simple Mantel tests using two matrices, we used partial Mantel tests to tease apart the pure effects of hydrological, environmental, and spatial variables on biotic matrices, and the significance was assessed using 9,999 permutations, as described above. Mantel and partial Mantel tests were run using functions *mantel* and *mantel.partial*, respectively, in R package *vegan* (version 2.4‐2).

## 3 RESULTS

### 3.1 Variability of abiotic factors

During the sampling period, river reaches of the study area (Figure 1) varied widely in water quality and habitat characteristics and the main abiotic variables are summarized in Table 1. For example, water temperature (WT) ranged from 0.20 to 8.40°C (mean: 5.69°C), pH ranged from 6.74 to 9.73 (mean: 7.49), total phosphorus (TP) averaged 0.22 mg/L (0.06–0.63 mg/L), and ammonium‐nitrogen (NH_4_‐N) ranged from 0.03 to 1.43 mg/L (mean: 0.31 mg/L), while total suspended particulates (TSP) averaged 12.08 mg/L (2.60–46.28 mg/L). Land use in the catchment was mainly open canopy and dominated by high agricultural land of 51.83% (15.04–79.65%), while forest cover was low (mean coverage was 5.71% ranging from 0.86 to 15.13%). Due to a heavy rainfall event during the sampling period, hydrological variables varied greatly among the sampling sites. For instance, flow velocity (VELO) ranged from 0 to 10.24 m/s with an average of 0.98 m/s, discharge (Hv01) ranged from 0.01 to 18.30 m^3^/s with a mean of 2.27 m^3^/s, while skewness of flows (Hv12, Hv13, Hv20, Hv21, and Hv36), low flood pulse count (Hv40), high flood pulse count (Hv45), and change rates of flows (Hv54 and Hv55) also showed large ranges (for details see Table 1). In addition, the spatial variables showed a small variation with latitude ranging from 54.51 to 54.74°N and longitude from 9.27 to 9.67°E, which was due to the relative small catchment of Treene (481 km^2^).

### 3.2 Drivers of traits and species composition

**Figure 2:**
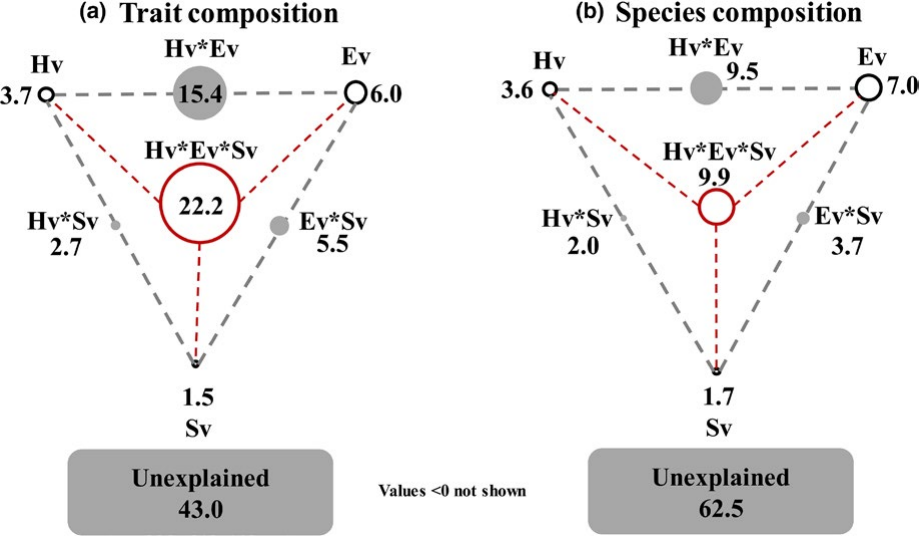
Contributions of the hydrological (Hv), environmental (Ev), and spatial variables (Sv) to the variances in trait (a) and species composition (b). Each diagram represents a given biological variation partitioned into the pure effects of Hv, Ev, and Sv (i.e., when removing the variations caused by other two factors), interaction between any two variables (Hv*Ev, Hv*Sv, and Ev*Sv), interaction of all three factors (indicated by red circle), and unexplained variation (total variation = 100). The geometric areas of circles were proportional to the respective percentages of explained variation. More details on the selected variables are shown in Table 2

In the RDA analysis for trait composition (Tr), hydrological (Hv), environmental (Ev), and spatial variables (Sv) all showed significant relationships with trait composition (by *anova* function in R package *vegan*, Table 2). Five Hv, nine Ev, and seven Sv variables were selected by forward selection. According to variation partitioning analysis, the three sets could explain 57.0% variation of trait composition (Figure 2a). The pure effects of Hv (3.7%) and Ev (6.0%) accounted for larger parts than the pure effect of Sv (1.5%), while the joint effect of Hv, Ev, and Sv was the largest with 22.2%.

**Table 2:** Results of forward selection of hydrological variables (Hv), environmental variables (Ev), and spatial variables (Sv) for trait (Tr) and species (Sp) composition, respectively

Trait composition (Tr)				Species composition (Sp)			
Variables	Adj*R* ^2^Cum	*F*	*p*	Variables	Adj*R* ^2^Cum	*F*	*p*
Hv***				Hv***			
Hv21	0.23	17.88	.001	Hv21	0.15	11.29	.001
Hv40	0.35	12.20	.001	Hv40	0.22	6.36	.001
Hv55	0.39	4.53	.004	Hv55	0.25	2.83	.008
Hv45	0.42	3.93	.011	Hv45	0.28	3.35	.003
Hv36	0.44	2.75	.031	Hv36	0.29	2.07	.036
Ev***				Ev***			
PO_4_	0.14	10.17	.001	SO_4_	0.06	4.45	.001
TP	0.33	16.93	.001	UR	0.10	3.55	.001
SO_4_	0.36	3.93	.003	WPAS	0.13	2.98	.001
PH	0.39	3.46	.004	WATR	0.15	2.44	.005
WT	0.41	3.39	.005	PO_4_	0.17	2.33	.008
DTC	0.44	3.25	.011	TP	0.22	4.53	.001
WPAS	0.46	3.76	.003	PH	0.24	2.51	.004
FRST	0.48	2.35	.040	DTC	0.26	2.37	.003
NO_2_	0.49	2.29	.043	WT	0.28	2.08	.009
				NH_4_	0.29	1.83	.024
				FRST	0.30	1.66	.037
Sv***				Sv*			
PCNM6	0.09	6.84	.001	PCNM6	0.05	4.02	.001
PCNM7	0.17	6.56	.001	PCNM7	0.08	2.86	.004
PCNM3	0.22	4.52	.003	PCNM3	0.10	2.59	.016
PCNM10	0.25	3.29	.020	PCNM10	0.12	2.24	.008
X	0.28	2.74	.036	X	0.14	2.26	.010
PCNM11	0.30	2.54	.039	PCNM1	0.17	2.78	.002
Y	0.32	2.79	.023				

The selected variables are in the order in which they were selected in the forward selection procedure. Adj*R* ^2^Cum (cumulative adjusted *R* ^2^), *F*, and *p* values are shown. All selected variables show no significant multicollinearity (with variance inflation factor VIF < 10, by *vif.cca* function in R package *vegan*). Codes of variables are as in Table 1. Significance was expressed as *p < .05, **p < .01, ***p < .001 (by *anova* function in R package *vegan*).

Similarly, in the CCA analysis for species composition (Sp), Hv, Ev, and Sv all showed significant relationships with species composition (by *anova* function in R package *vegan*) and five Hv, 11 Ev, and six Sv variables were selected by forward selection (Table 2). Variation partitioning indicated that the three sets explained only 37.5% variation of species composition. The variation purely explained by Hv, Ev, and Sv was 3.6%, 7.0%, and 1.7%, respectively, while the shared fraction was 9.9% (Figure 2b).

In general, the joint contribution by Hv and Ev (Hv*Ev) (Tr: 15.4%, Sp: 9.5%) was higher than those by Hv*Sv (Tr: 2.7%, Sp: 2.0%) and Ev*Sv (Tr: 5.5%, Sp: 3.7%) (Figure 2). The unexplained fraction of trait composition (43.0%) was lower than for species composition (62.5%) (Figure 2). However, the variation partitioning (Figure 2) showed that both trait and species composition were less dependent on spatial factors, rejecting our third hypothesis.

### 3.3 Main drivers of traits and species‐based beta diversities

**Figure 3:**
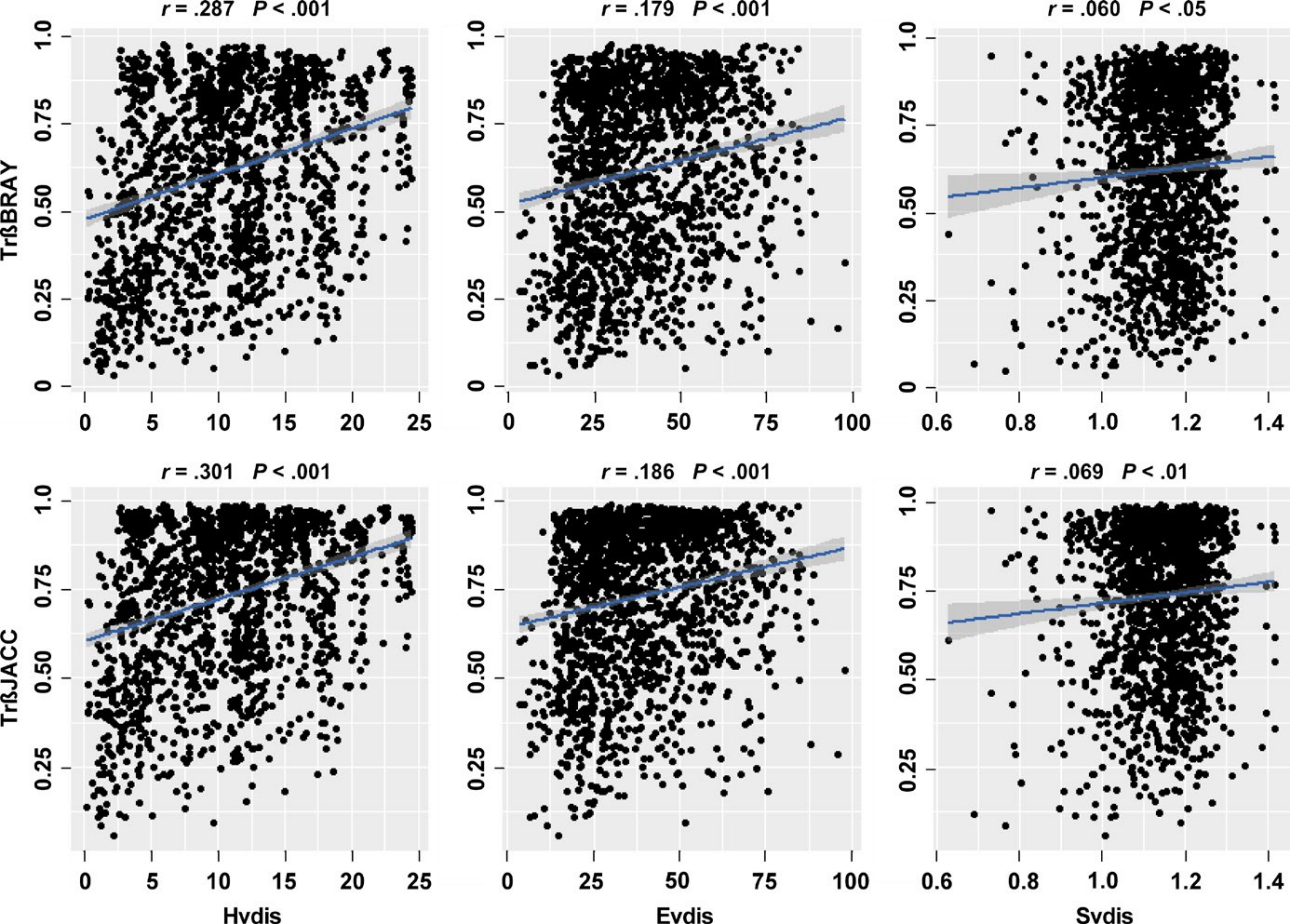
Relationship between trait dissimilarities (Bray–Curtis and Jaccard: Trß_BRAY_ and Trß_JACC_) and hydrological (Hvdis), environmental (Evdis), and spatial Euclidean distances (Svdis). The relationships were statistically significant according to the Mantel test (9,999 permutations, *p* < .05, see Table 3). Regression lines based on linear models are shown by solid blue lines, and shaded gray area indicates 95% confidence interval of the fit

Mantel tests showed that trait dissimilarities (i.e., beta diversities) based on both Bray–Curtis and Jaccard indices (Trß_BRAY_ and Trß_JACC_) increased significantly with hydrological (Hvdis), environmental (Evdis), and spatial distances (Svdis) (Figure 3, Table 3). The relationships between trait dissimilarities (Trß_BRAY_ and Trß_JACC_) and hydrological distances (Hvdis) were consistently stronger than the relationships with environmental distances (Evdis), while the weakest relationships were with spatial distances (Svdis) (Figure 3). Based on partial Mantel tests, the pure effects of hydrological and environmental distances on trait dissimilarities were significant using both indices, whereas the pure effect of spatial distance was nonsignificant using both indices (Table 3).

**Table 3:** Results of Mantel and partial Mantel test for the correlation between ß diversities for traits (Tr) and species (Sp) (Bray–Curtis and Jaccard: Trß_BRAY,_ Trß_JACC,_ Spß_BRAY_, and Spß_JACC_) and hydrological (Hvdis), environmental (Evdis), and spatial Euclidean distances (Svdis)

Index	Hvdis	Evdis	Svdis	Hvdis^a^	Evdis^a^	Svdis^a^
Trß_BRAY_	0.287***	0.179***	0.060*	0.311**	0.218**	0.016
Trß_JACC_	0.301***	0.186***	0.069**	0.327**	0.228**	0.023
Spß_BRAY_	0.218***	0.188***	0.032	0.242**	0.216**	−0.013
Spß_JACC_	0.224***	0.179***	0.039	0.247**	0.207**	−0.004

a The pure effect while controlling for the other two distances.

*p < .05, **p < .01, ***p < .001.

**Figure 4:**
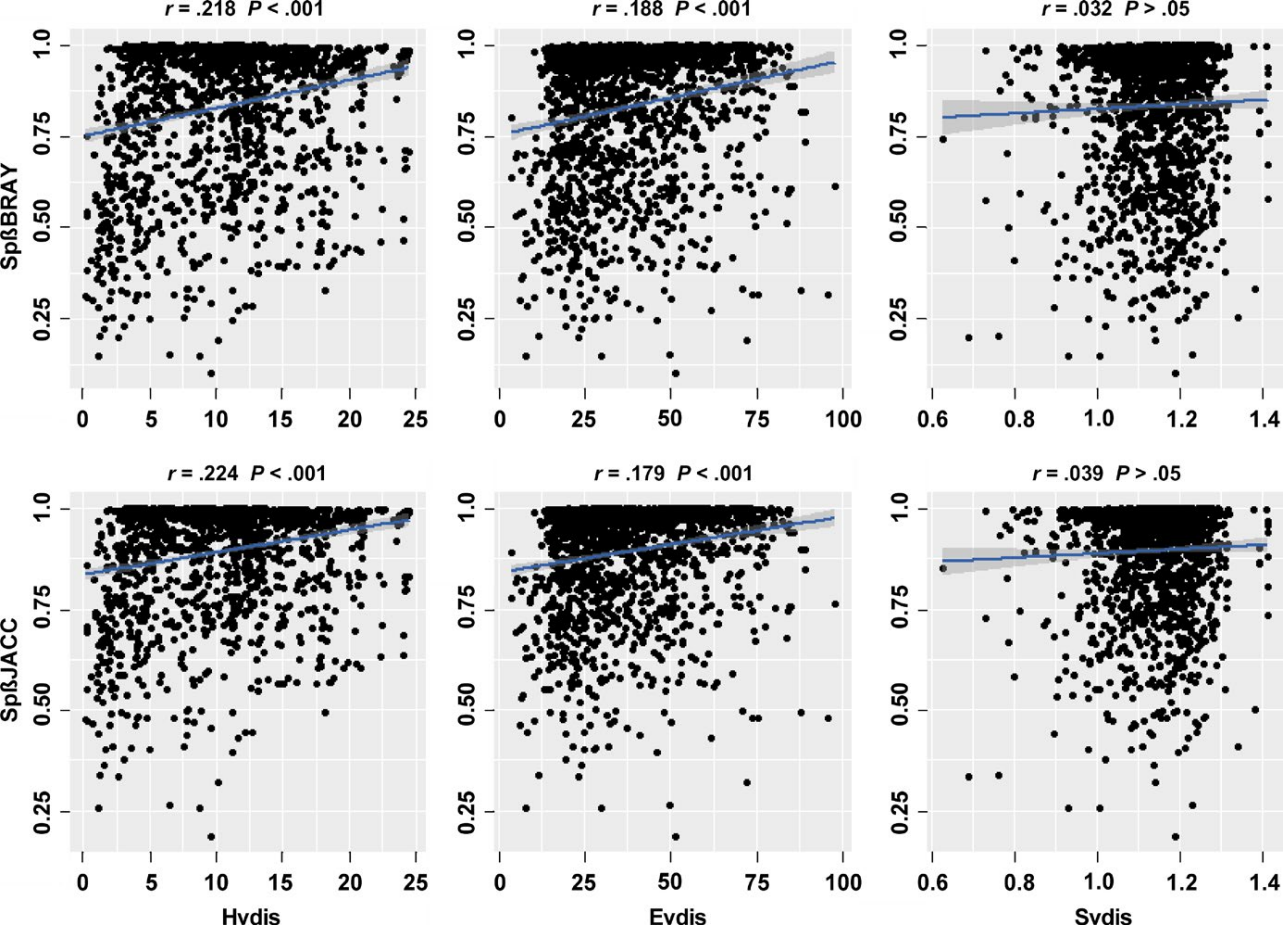
Relationship between species dissimilarities (Bray–Curtis and Jaccard: Spß_BRAY_ and Spß_JACC_) and hydrological (Hvdis), environmental (Evdis), and spatial Euclidean distances (Svdis). The relationships were statistically significant according to the Mantel test (9,999 permutations, *p* < .05, see Table 3). Regression lines based on linear models are shown by solid blue lines, and shaded gray area indicates 95% confidence interval of the fit

As for species dissimilarities based on both Bray–Curtis and Jaccard indices (Spß_BRAY_ and Spß_JACC_), similar results were found (Figure 4, Table 3). The pairwise species compositional dissimilarities (Spß_BRAY_ and Spß_JACC_) significantly increased with the corresponding changes in hydrological (Hvdis) and environmental distances (Evdis). Further, the relationships between species dissimilarities (Trß_BRAY_ and Trß_JACC_) and hydrological distances (Hvdis) were consistently stronger than the relationships with environmental distances (Evdis) (Table 3). In contrast, there was no significant spatial distance–decay for both indices (*p* > .05) (Figure 4). According to partial Mantel tests, the pure effect of spatial distance was nonsignificant, while the pure effects of hydrological and environmental distances on species dissimilarities were significant using both indices (Table 3).

## 4 DISCUSSION

One of the long‐standing tasks in ecology is to explore the factors controlling the abundance and distribution patterns of aquatic organisms and the causes underlying these patterns. Although the relationship between algae community and abiotic factors (e.g., resources and disturbances), as well as grazers, has been intensively investigated, the relative roles of different factors to algal variations remain controversial (Wu et al., 2011). For example, some studies found that the geographical topography (e.g., altitude, latitude, and longitude) and climate were the dominant factors regulating algae variation (Bae et al., 2014; Tang, Niu, et al., 2013; Tang, Wu, et al., 2013; Wu et al., 2014). In contrast, local environmental variables (e.g., substrate composition, sediments, nutrients, oxygen contents, and biointeraction) were often considered to be the main regulating factors (Bae et al., 2014; Bussi et al., 2016). Besides, previous studies have rarely taken hydrological variables into consideration. This might be due to the fact that acquisition of accurate hydrological variables needs long‐term discharge data at different sampling sites, which is often time‐consuming (e.g., the measurement of discharge). Obtaining data from field hydrological stations is an alternative way, but it is normally impossible for every sampling site because of the limited numbers of hydrological stations, for instance only six stations in our catchment with 59 sampling sites (Figure 1). At this situation, a well‐proven hydrological modeling would be a good choice as it enables to predict long‐term discharge variations at different sampling sites, as the case in our study area.

In this study, we used hydrological modeling to obtain hydrological data for 59 sites and, as expected, found that the hydrological variables, for example, skewness of flow (Hv21, Hv36), flood pulse count (Hv40, Hv45), and change rate of flow (Hv55), were the most important factors affecting both trait and species composition. Hydrological conditions are general factors that determine the physical habitat conditions and affect (directly or indirectly) many other environmental variables that are key factors in pelagic algae community development, such as nutrient delivery, sediment transportation, residence time, disturbance intensity, temperature, light availability, and dissolved oxygen. That was also the reason why hydrological variables showed a higher shared effect with local environmental variables (Figure 2). With the large number of available hydrological metrics in use today, flow variabilities such as the magnitude, frequency, duration, timing, and rate of change in flows were the most important factors regulating ecological processes in aquatic ecosystems (Bhat, Jacobs, Hatfield, & Graham, 2010). A previous study on the relations among 83 hydrological metrics and changes in algal communities of the United States was consistent with our study and demonstrated the importance of hydrological variables to the variance of specific algal community metrics (Steuer, Stensvold, & Gregory, 2010). Moreover, recent studies (Qu et al., 2018; Wu et al., 2016), which were in line with our finding, also found that hydrological conditions played an important role in temporal variations of pelagic algae communities. Skewness of flows was found to be one of the most consistently dominant indices across all stream types and may be a particularly important measure of flow condition for certain riverine taxa (Olden & Poff, 2003), for example, annual skewness of the flow has been linked to fish mobility and colonizing ability (Puckridge, Sheldon, Walker, & Boulton, 1998). High‐flow event frequency (e.g., flood pulse count and change rate of flow), which was found to be transferable across stream type, was the most ecologically relevant hydrological condition metrics. Previous studies with the aim of characterizing the response of phytoplankton to high‐flow events have indicated the importance of flow events in driving the patterns of phytoplankton distribution (Cook, Holland, & Longmore, 2010; Saeck, Hadwen, Rissik, O'Brien, & Burford, 2013). However, how does individual hydrological variable affect the pelagic algae composition and diversity was still less investigated so far and a possible reason was that few studies have the necessary temporal and spatial resolution to fully characterize these effects. This also remains a need to identify their individual or joint impacts and associated mechanisms in the future studies by means of intensive field campaigns or indoor mesocosms. Furthermore, prompted by the importance of hydrological variables to algal communities, we therefore advocate that planning for long‐term monitoring and biodiversity conservation or restoration should include hydrological variables.

Besides, interdisciplinary collaboration between ecology and hydrology warrants further attention as it can advance our knowledge in understanding the aquatic organisms in relation to abiotic factors, particularly the hydrological conditions. This is in line with combining measurement campaigns with coupled abiotic–biotic modeling with the aim to improve the abundance/occurrence of biota and their ecohydrological drivers. As shown in this study, spatially distributed hydrological model studies allow an identification of hydrological conditions that can be used to describe the abundance and occurrence of biota. The results of hydrological models can be used both as input for ecological habitat models (Guse, Kail, et al. 2015; Guse, Pfannerstill, et al., 2015), to describe the habitat of different biota (Kiesel, Hering, Schmalz, & Fohrer, 2009) and for consecutive data analysis based on the model results as shown here and a recent study (Kiesel et al., 2017).

Our results also demonstrated that the spatial factors were less important than local hydrological and environmental variables for both trait and species composition (Figure 2). For lowland rivers, the question about where do riverine pelagic algae come from is an important issue and has long been debated as it directly determines the suitability of pelagic algae‐based bioassessment, which were more and more frequently used at lowland catchments (Wu, Schmalz, & Fohrer, 2012). Historically, it was believed that there was no true riverine plankton and the pelagic algae found in rivers were brought from either upstream lentic water bodies or the benthos (Hötzel & Croome, 1999). Obviously, if this view was right, the riverine pelagic algae were not suitable as a bioindicator because they were flushed or drifted and not adapted to the local environmental habitats. As a consequence, riverine pelagic algae were less used for biomonitoring than other communities, such as periphyton and benthic invertebrates. However, recent studies (Centis, Tolotti, & Salmaso, 2010; Wu et al., 2011) have argued that the idea of benthic diatom communities being the source of the riverine pelagic algae may be too simplistic, and they believed that planktonic algal species do reproduce within rivers and many species develop substantial populations in situ. Disentangling the relative roles of local and spatial variables on spatial pattern of the community is a promising way to understand the source of pelagic algae communities. Based on the metacommunity theory (Heino et al., 2015), the observed community at a certain point is shaped by two broad categories of effects—local and regional (i.e., spatial) effects. Local effects are largely due to environmental constraints or species interactions, while spatial effects are driven by the flux of organisms from the regional species pool (Brown & Swan, 2010). Our results in this study showed that the pelagic community in Treene catchment was more affected by local effects (e.g., local hydrological and environmental variables) than spatial effects as indicated by spatial variables (Figure 2). These findings supported the recent studies (Qu et al., 2018; Wu et al., 2011) and further emphasized the suitability of lowland pelagic algae as bioindicator for local habitat changes. Nevertheless, factors such as interaction between organisms (niche competition), dispersal ability, and species evolution, which were not considered in this study, may have reduced the explainable variations. Furthermore, the relative importance of different factors may vary among different regions and might depend on the spatial extent of the study area.

Another interesting finding showed that trait and species composition were both less dependent on spatial factors (Figure 2), which contradicted our third hypothesis. As an alternative to species‐based approaches, use of trait‐based approaches in biomonitoring has been advocated in recent years, in particular because of the demand of mechanistic understanding of biological responses (Baattrup‐Pedersen, Göthe, Riis, Andersen, & Larsen, 2017). Based on previous studies (B‐Béres et al., 2016; Lange et al., 2016; Passy, 2007; Soininen et al., 2016), trait composition would track local environment gradients better than species composition and was less dependent on historic (i.e., spatial) factors, making them better suitable for research on global environmental change (Soininen et al., 2016). Nevertheless, our finding was rather unexpected compared to a recent similar study (Soininen et al., 2016). These differences between findings may be related to the spatial scale of studied areas. In comparison with a single catchment of this study, the previous research compared the trait and species composition at a global scale (Soininen et al., 2016). Generally, for species distribution, the importance of spatial effects increased with geographical distance as dispersal limitation, and at large scales, spatial effects might outperform local environmental effects (Heino et al., 2010; Wu et al., 2014). Therefore, further comparisons between trait and species composition in relation to different factors at multispatial scales are greatly needed.

Mantel tests suggested that the importance accounting for the among‐site differences in species and trait‐based beta diversities was as follows: hydrological variables > environmental filtering, without effects of historic (spatial) factors. Identifying mechanisms underlying the spatial patterns of biodiversity is another important task in community ecology, as these are fundamental to the appropriate biodiversity conservation and restoration (Myers et al., 2000; Wang, Pan, et al., 2016). Focusing on pelagic algae in a catchment with short geographical distances and incorporating multiple factors enabled the disentanglement of pure hydrological, environmental, and spatial gradients in our study. Our results revealed a clear distance–decay of community dissimilarity with increasing hydrological and environmental distances (Figures 3 and 4, Table 3). However, the relative roles of different distance matrices showed considerable variability, for instance, the importance of hydrological distance was consistently stronger than environmental distance, while importance of spatial distance was the lowest (or even nonsignificant). A key implication of our findings for biodiversity conservation is that maintaining the instream flow regime and keeping various habitats among rivers are of vital importance.

In conclusion, the present study has revealed the clear important role of flow regime (indicated by hydrological variables) in structuring riverine algae communities and beta diversity patterns, which, in particular for beta diversities, has outperformed with local environmental variables and spatial factors. Our findings further emphasize the fundamental importance of considering hydrological variables, particularly when planning for long‐term monitoring and biodiversity conservation or restoration. Although both trait and species composition showed significant correlations with hydrological, environmental, and spatial variables, respectively, higher variation of trait composition (57.0%) than species composition (37.5%) was caught by these factors. This emphasizes the merit of applying traits for biomonitoring and management of freshwater ecosystems. As our sampling covered only one catchment, we admit that the generality of these findings will be assessed later by other investigations in different systems. We also advocate that researchers should consider multispatial and temporal scales explicitly in studies of biodiversity conservation, as pattern may change with study scales (Li, Chung, Bae, Kwon, & Park, 2012; Soininen, McDonald, & Hillebrand, 2007; Tang, Jia, Jiang, & Cai, 2016).

## CONFLICT OF INTEREST

The authors declare no conflict of interest.

## AUTHOR CONTRIBUTION

N.W., T.R., and N.F. developed the basic ideas. N.W., Y.Q., and K.M. performed the sampling collection and processing. Y.Q. and K.M. identified the algae samples. K.M. provided the land use data. B.G. ran the SWAT modeling. Y.Q. calculated the hydrological variables. S.T. calculated the trait composition. N.W. performed the data analyses and led the main writing. B.G. wrote the description of SWAT modeling. All authors have seen the manuscript before submission.

## DATA ACCESSIBILITY

All data have been uploaded as Appendices S1–S5.
