# Supplementary material for: Hydrological and environmental variables outperform spatial factors in structuring species, trait composition, and beta diversity of pelagic algae
Source: Ecol Evol. 2018 Feb 14;8(5):2947–61. doi: 10.1002/ece3.3903 (PMC5838050; doi:10.1002/ece3.3903)
Supplement: Supplementary file 1 [file ECE3-8-2947-s001.docx]

# SUPPORTING INFORMATION

# Hydrological and environmental variables outperform spatial factors in structuring species, trait composition and beta diversity of pelagic algae

Naicheng Wu ^1,2,3*^, Yueming Qu^1^, Björn Guse**^1,5^**, Kristė Makarevičiūtė^4^**,** Szewing To^1^**,** Tenna Riis^3^ and Nicola Fohrer^1^

*^1^Department of Hydrology and Water Resources Management, Institute for Natural Resource Conservation, Kiel University, 24118 Kiel, Germany*

*^2^Aarhus Institute of Advanced Studies, Aarhus University, Høegh-Guldbergs Gade 6B, 8000 Aarhus C, Denmark*

*^3^Department of Bioscience, Aarhus University, Ole Worms Allé 1, 8000 Aarhus C, Denmark*

*^4^Helmholtz Centre for Ocean Research Kiel (GEOMAR), DüsternbrookerWeg 20, 24105 Kiel, Germany*

*^5^GFZ German Research Centre for Geosciences, Section 5.4 Hydrology, Potsdam, Germany*

Appendix S1. Algal traits, their categories, and abbreviations used in this study.

| Traits | Categories | Abbreviations |
| --- | --- | --- |
|  |  |  |
| 1. Cell size  (Berthon, Bouchez & Rimet 2011; Rimet & Bouchez 2012) | Pico (< 5 µm^3^) | BioVol_C0 |
|  | Nano (5-100 µm^3^) | BioVol_C1 |
|  | Micro (100-300 µm^3^) | BioVol_C2 |
|  | Meso (300-600 µm^3^) | BioVol_C3 |
|  | Macro (600-1500 µm^3^) | BioVol_C4 |
|  | Large (> 1500 µm^3^) | BioVol_C5 |
| 2. Guild  (Passy 2007; Rimet & Bouchez 2012) | Low profile | LowPro |
|  | High profile | HigPro |
|  | Motile taxa | MotTax |
|  | Planktonic taxa | PlaTax |
| 3. Life form  (Ferragut & Campos Bicudo 2010) | Colonial | LifFor_col |
|  | Filamentous | LifFor_fil |
|  | Flagellate | LifFor_fla |
|  | Unicellular | LifFor_uni |
| 4. Eco-morphology | Guild +Cell size*  (B-Béres *et al.* 2016) | LC0, LC1, LC2, LC3, LC4, LC5 |
|  |  | HC0, HC1, HC2, HC3, HC4, HC5 |
|  |  | MC0, MC1, MC2, MC3, MC4, MC5 |
|  |  | PC0, PC1, PC2, PC3, PC4, PC5 |
|  | Life form +Cell size** | ColC0, ColC1, ColC2, ColC3, ColC4, ColC5 |
|  |  | FilC0, FilC1, FilC2, FilC3, FilC4, FilC5 |
|  |  | FlaC0, FlaC1, FlaC2, FlaC3, FlaC4, FlaC5 |
|  |  | UniC0, UniC1, UniC2, UniC3, UniC4, UniC5 |
| 5. Nitrogen fixation  (Stancheva *et al.* 2013) | Yes (1) or no (0) | NitFix_1 |
| 6. Reproductive strategies  (Biggs, Stevensen & Lowe 1998) | Fission | RepStr_fis |
|  | Fragmentation | RepStr_fra |
| 7. Spore formation  (Agrawal 2009; Lange, Townsend & Matthaei 2016) | No spore formation | SpoFor_non |
|  | Zoospores | SpoFor_zoo |
|  | Akinetes | SpoFor_aki |
|  | Oospores and zygospores | SpoFor_oos.zyg |

*A simple combination between 4 guilds and 6 cell size classes, resulting in 24 combinations, adapted from (B-Béres *et al.* 2016).

**A simple combination between 4 life forms and 6 cell size classes, resulting in 24 combinations, developed by this study.

Appendix S2. Description of the hydrological indices (Hv) used in this study (Olden & Poff 2003).

| Code | Unit | Description | Definition |
| --- | --- | --- | --- |
| **Magnitude of flow events** | | |  |
| Hv01 | m^3^/s | Discharge at the sample day | The discharge predicted by the SWAT model |
| Hv12 | - | Skewness of 3 days' ahead discharge (including the sampling day) | (Mean daily flow-median daily flow)/median daily flow |
| Hv13 | - | Skewness of 3 days' ahead discharge (excluding the sampling day) |  |
| Hv20 | - | Skewness of 7 days' ahead discharge (including the sampling day) |  |
| Hv21 | - | Skewness of 7 days' ahead discharge (excluding the sampling day) |  |
| Hv36 | - | Skewness of 30 days' ahead discharge (including the sampling day) |  |
| **Frequency of flow events** | | |  |
| Hv40 | d | Low flood pulse count in the past 14 days | Low flood pulses are defined as the number of days in which the flow drops below the 25^th^ percentile (low pulse) of all daily values for the time period (2010-2016). |
| Hv45 | d | High flood pulse count in the past 30 days | High flood pulses are defined as the number of days in which the flow rises above the 75^th^ percentile (high pulse) of all daily values for the time period (2010-2016). |
| **Rate of change in flow events** | | |  |
| Hv54 | - | Rate of change (i.e. slope) in the last 3 days before the sampling day | Mean rate of changes in flow from 1^st^ day to the 3^rd^ day |
| Hv55 | - | Rate of change (i.e. slope) in the last 7 days before the sampling day | Mean rate of changes in flow from 1^st^ day to the 7^th^ day |
| ***In situ* measurement** | | |  |
| VELO | m/s | Flow velocity at the sampling point | Measured *in situ* at the field |

Variables indicating significant multicollinearity (with variance inflation factor >10 and Spearman correlation coefficient >=0.75) are excluded.

Appendix S3. Pairwise Spearman correlation of hydrological variables (Hv). Variables indicating significant multicollinearity (with variance inflation factor >10 and Spearman correlation coefficient >=0.75) are excluded. Codes of variables are as in Table1.

|  | Hv01 | Hv12 | Hv13 | Hv20 | Hv21 | Hv36 | Hv40 | Hv45 | Hv54 | Hv55 | VELO |
| --- | --- | --- | --- | --- | --- | --- | --- | --- | --- | --- | --- |
| Hv01 | 1.000 | -0.202 | 0.215 | -0.304 | -0.274 | 0.062 | -0.608 | 0.667 | 0.700 | -0.418 | 0.380 |
| Hv12 | -0.202 | 1.000 | -0.202 | 0.097 | 0.136 | 0.161 | 0.081 | -0.256 | -0.046 | 0.039 | -0.071 |
| Hv13 | 0.215 | -0.202 | 1.000 | 0.282 | 0.094 | 0.383 | -0.039 | -0.103 | 0.261 | 0.152 | 0.160 |
| Hv20 | -0.304 | 0.097 | 0.282 | 1.000 | 0.745 | 0.219 | 0.102 | -0.510 | -0.167 | 0.508 | -0.031 |
| Hv21 | -0.274 | 0.136 | 0.094 | 0.745 | 1.000 | 0.266 | 0.179 | -0.395 | 0.125 | 0.703 | 0.003 |
| Hv36 | 0.062 | 0.161 | 0.383 | 0.219 | 0.266 | 1.000 | -0.129 | -0.198 | 0.258 | 0.334 | 0.031 |
| Hv40 | -0.608 | 0.081 | -0.039 | 0.102 | 0.179 | -0.129 | 1.000 | -0.642 | -0.434 | 0.245 | -0.307 |
| Hv45 | 0.667 | -0.256 | -0.103 | -0.510 | -0.395 | -0.198 | -0.642 | 1.000 | 0.412 | -0.465 | 0.338 |
| Hv54 | 0.700 | -0.046 | 0.261 | -0.167 | 0.125 | 0.258 | -0.434 | 0.412 | 1.000 | -0.036 | 0.329 |
| Hv55 | -0.418 | 0.039 | 0.152 | 0.508 | 0.703 | 0.334 | 0.245 | -0.465 | -0.036 | 1.000 | 0.065 |
| VELO | 0.380 | -0.071 | 0.160 | -0.031 | 0.003 | 0.031 | -0.307 | 0.338 | 0.329 | 0.065 | 1.000 |

Appendix S4. Pairwise Spearman correlation of environmental variables (Ev). Variables indicating significant multicollinearity (with variance inflation factor >10 and Spearman correlation coefficient >=0.75) are excluded. Codes of variables are as in Table1.


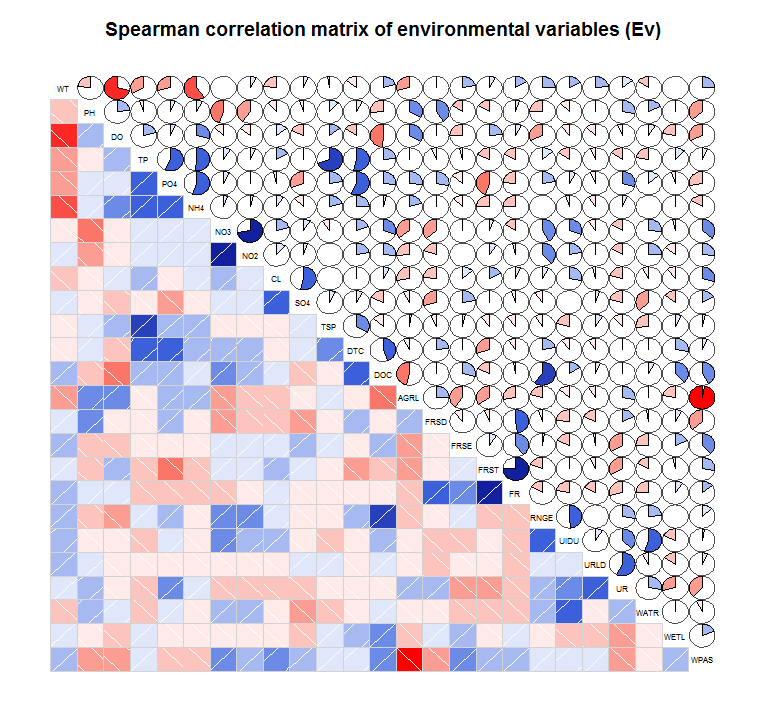


Appendix S5. Pairwise Spearman correlation of spatial variables (Sv). X = Latitude, Y = Longitude, numbers 1-37 indicate PCNM1 – PCNM37.


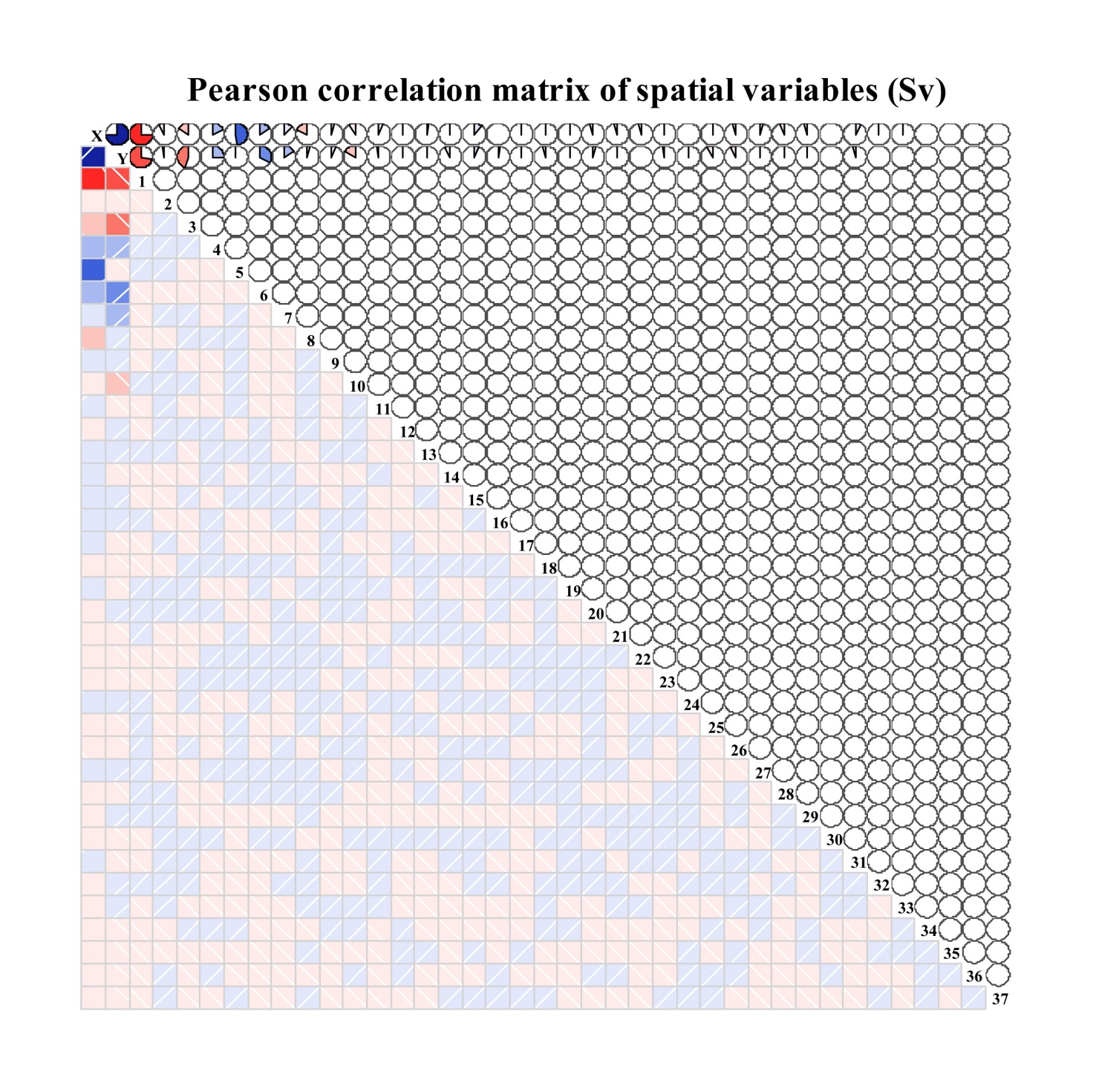


**REFERENCES**

Agrawal, S.C. (2009) Factors affecting spore germination in algae — review. *Folia Microbiologica,* **54,** 273-302.

B-Béres, V., Lukács, Á., Török, P., Kókai, Z., Novák, Z., T-Krasznai, E., Tóthmérész, B. & Bácsi, I. (2016) Combined eco-morphological functional groups are reliable indicators of colonisation processes of benthic diatom assemblages in a lowland stream. *Ecological Indicators,* **64,** 31-38.

Berthon, V., Bouchez, A. & Rimet, F. (2011) Using diatom life-forms and ecological guilds to assess organic pollution and trophic level in rivers: a case study of rivers in south-eastern France. *Hydrobiologia,* **673,** 259-271.

Biggs, B.J.F., Stevensen, R.J. & Lowe, R.L. (1998) A habitat matrix conceptual model for stream periphyton. *Archiv für Hydrobiologie,* **143,** 21-56.

Ferragut, C. & Campos Bicudo, D. (2010) Periphytic algal community adaptive strategies in N and P enriched experiments in a tropical oligotrophic reservoir. *Hydrobiologia,* **646,** 295-309.

Lange, K., Townsend, C.R. & Matthaei, C.D. (2016) A trait-based framework for stream algal communities. *Ecology and Evolution,* **6,** 23-36.

Olden, J.D. & Poff, N.L. (2003) Redundancy and the choice of hydrologic indices for characterizing streamflow regimes. *River Research and Applications,* **19,** 101-121.

Passy, S.I. (2007) Diatom ecological guilds display distinct and predictable behavior along nutrient and disturbance gradients in running waters. *Aquatic Botany,* **86,** 171-178.

Rimet, F. & Bouchez, A. (2012) Life-forms, cell-sizes and ecological guilds of diatoms in European rivers. *Knowledge and Management of Aquatic Ecosystems,* **406,** 01-14.

Stancheva, R., Sheath, R.G., Read, B.A., McArthur, K.D., Schroepfer, C., Kociolek, J.P. & Fetscher, A.E. (2013) Nitrogen-fixing cyanobacteria (free-living and diatom endosymbionts): their use in southern California stream bioassessment. *Hydrobiologia,* **720,** 111-127.
